# Fathers' experiences, views and perspectives of childbirth attendance: A qualitative evidence synthesis protocol

**DOI:** 10.12688/hrbopenres.13879.1

**Published:** 2024-06-06

**Authors:** Nazia AlAmri, Sunita Panda, Valerie Smith

**Affiliations:** 1 School of Nursing and Midwifery, The University of Dublin Trinity College, Dublin, Leinster, Ireland; 2 School of Nursing, Midwifery and Health Systems, University College Dublin, Dublin, Leinster, Ireland

**Keywords:** Fathers, Childbirth, Attendance

## Abstract

**Background:**

The involvement of husbands and male partners in childbirth no longer ceases at conception and pregnancy, rather fathers wish to be more involved in supporting their partners during childbirth. This aligns with the World Health Organization's (WHO) call for promoting male participation in childbirth, emphasising the benefits of support and the positive impact attending childbirth has for husbands/partners, and their maternal partner. This knowledge has led to global initiatives promoting "humanised" birth and a family-approach. To gain in-depth insight and understanding of childbirth attendance from the perspectives of fathers, a qualitative evidence synthesis is proposed.

**Methods:**

To explore fathers' experiences, views, and perspectives of childbirth attendance. All studies that used qualitative methodologies to explore the phenomenon of interest will be included. MEDLINE, CINAHL, PsycINFO, MIDIRS, Web of Science, and Google Scholar will be systematically searched from their dates of inception to present, supplemented by a search for grey literature and a search of the reference list of included studies. Peer Review of Electronic Search Strategies (PRESS) will be used to ensure the comprehensiveness of the search strategy. Methodological quality assessment of included studies, using The Critical Appraisal Skills Programme assessment tool, and will be extracted from the included studies by two reviewers independently using a standard data extraction form. Thomas and Harden’s three-stage approach will be used to thematically synthesise the data: coding of data, developing descriptive themes, and generation of analytical themes. The Grading of Recommendations Assessment, Development, and Evaluation-Confidence in the Evidence from Reviews of Qualitative research (GRADE-CERQual confidence in the synthesised findings Comprehensive insight and understanding of fathers’ perspectives of childbirth attendance will be ascertained

**PROSPERO Registration No:**

CRD42023470902

## Introduction

Historically, childbirth was considered a domain exclusive to women. Societal expectations and legal restrictions were some of the main reasons that largely excluded men from the childbirth process until the mid-20th century ( Leathers & Esterberg, 2004). Up until the 1970s it was not the norm for fathers to attend childbirth. When fathers were gradually facilitated to attend birth, there was initial resistance from both the public and healthcare institutions that prevented men’s attendance in the birthing room ( Davis-Floyd, 2009; Leap & Murphy, 1994). Chapman (1991) speculated that concerns over increased infection rates and potential legal implications such as malpractice or negligence contributed to the reluctance to allow men in the labour room. Additionally, it was argued that fathers might struggle to cope with the physiological aspects of childbirth, potentially leading them to panic, resulting in increased workload for healthcare professionals ( Chapman, 1991; Draper, 1997).

In 1985, the World Health Organisation (WHO), reported that only 13 countries facilitated fathers to be present during birth. In the same year, WHO advocated for unrestricted access to a chosen person or family member to support and ensure the well-being of the mother ( WHO, 1985). Subsequently, in 2015, the WHO established guidelines to encourage men’s involvement in the birthing process ( WHO, 2015). Furthermore, evolving societal and professional attitudes towards fatherhood and family-centered care have prompted research efforts and increased awareness among healthcare professionals ( Lamb *et al.*, 2004). This shift recognises birth as a shared experience extending beyond the traditional mother-baby dyad, fostering a more inclusive and supportive birthing environment. A growing body of research highlights the benefits of support during labour and birth such as increased emotional attachment, reduced post-birth trauma, and improved outcomes for both mother and baby ( Hodnett *et al.,* 2014; Moore & Anderson, 2017). Fathers who were present at birth reported feeling more confident and prepared for their role as fathers and experienced a stronger emotional bond with their newborn ( Johnson *et al.*, 2012). Furthermore, women reported experiencing reduced anxiety and pain, and felt more satisfied with the birth when their husbands were involved or supported them during birth ( Grøtela *et al.*, 2018). Alternatively, studies suggest that women who did not receive or lacked support from their partner’s during childbirth had increased maternal stress and a higher risk of postpartum depression ( Beck, 2009; Schmied *et al.,* 2018).

 Although no country-wide national policies that restrict men from attending childbirth appear to exist, some cultures or religious sects may have traditions that discourage men from being present during childbirth or beliefs may influence childbirth practices, limiting the fathers’ involvement ( Mohammad-Ali *et al.,* 2022). Similar trends are observed in some Middle Eastern regions such as Bahrain and Saudi Arabia ( Mathews & Macintyre, 2018). As part of a wider body of research, that aims to explore fathers’ views and perspectives of childbirth attendance in the Kingdom of Bahrain, we undertook this qualitative evidence synthesis (QES) to first gain insight and understanding of fathers’ perspectives in the global context. Although two other qualitative reviews of fathers’ experiences of childbirth were identified, these reviews focused on factors that influenced fathers’ experiences ( McNab *et al.,* 2022; Mprah *et al.,* 2023). The current QES, contrastingly, will focus explicitly on the notion of childbirth attendance, thus bringing together the perspectives of fathers who have and may never have attended childbirth. The findings of this QES, while informing aspects of the wider study (e.g., semi-structured interview guide), will also have broader international practice and policy implications as it will identify potential areas for improvement in clinical practice and provide valuable insight to inform and support institutional guidelines.

### Aim

To provide a protocol for a QES that will synthesise the available qualitative evidence on fathers’ experiences, views, and perspectives of childbirth attendance.

To ensure transparency and methodological rigor, the proposed QES has been registered with the International Prospective Register of Systematic Reviews (PROSPERO: CRD42023470902, https://www.crd.york.ac.uk/prospero/display_record.php?RecordID=470902). The protocol report adheres to the Preferred Reporting Items for Systematic Reviews and Meta-Analyses Protocols (PRISMA-P) guidelines (Supplementary file 1: https://osf.io/9wscm/).

### Inclusion criteria

The inclusion criteria were defined using the SPIDER acronym ( Cooke *et al.,* 2012). Studies will be considered for inclusion if they report on:

**S**ample: Cis-gender male fathers above 18 years of age.

The QES will include all fathers regardless of their previous experience of attending childbirth. The sample population is limited to Cis-gender males to enable the application of the findings into the context of the wider study within Bahrain. Bahrain, being a culturally Islamic country, by law only recognises parenthood within a heterosexual marriage.

**P**henomenon of **I**nterest: All studies reporting on fathers’ experiences, views or perspectives of father attendance during labour and birth will be included.

**D**esign: All published and unpublished studies using qualitative methodologies such as focus groups, interviews or narrative research, irrespective of date conducted, or country of origin will be included.

**E**valuation of outcomes: Themes derived from the qualitative or narrative data that are representative of fathers’ views, perspectives, and experiences of childbirth attendance.

**R**esearch type: Primary qualitative studies (e.g., grounded theory, phenomenology, qualitative descriptive studies) that use recognised methods of qualitative data collection (e.g., interviews) and qualitative data synthesis methods. Survey designs with free-text response options will also be considered for inclusion if the available qualitative data provides sufficient depth and has been analysed formally using a structured approach (e.g. thematic analysis, content analysis, etc.). In case of mixed method studies, the qualitative component of the study where the participants' experiences are reported separately, will be included. Opinion pieces and commentaries from fathers on childbirth will be considered for inclusion if there is qualitative data of sufficient depth to contribute to data synthesis.

### Search strategy

Guided by SPIDER, search strings were developed for retrieving relevant literature. Within string search terms are combined using the OR Boolean operand, and each complete string combined then using the AND operand ( Table 1).

**Table 1.** Search terms.

Sample	Phenomena of Interest	Evaluation of outcomes	Research Type
Father* Husband* Spouse* Partner*	Parturition Birth Childbirth Delivery Pregnancy, antenatal, ante- natal, prenatal, antepartum, intrapartum, intra- natal, labour, labor, postnatal, post-natal, postpartum	attitude* view* perception* perspective* opinion* impact* experience* feeling* Belief	Qualitative Interview Mixed methods Hermeneutics Observation Narrative

A systematic search of the databases MEDLINE, CINAHL, PsycINFO, MIDIRS, Web of Science, and Google Scholar will be conducted from the database date of inception to the date the search is implemented. The search strings will be adapted for controlled vocabulary across the databases (Supplementary file 2: https://osf.io/9wscm/). Grey literature sources will include Open Grey, ProQuest Dissertations & Theses Abstract & Index, and Trinity's Access to Research Archive (TARA). The reference lists of included studies will be screened to identify potentially relevant additional studies that may not have been captured by the search of all other sources. To enhance methodological rigor, the search strategy was independently assessed by a topic expert not involved in the review using the Peer Review of Electronic Search Strategies (PRESS) checklist (Supplementary file 3: https://osf.io/9wscm/).

While the initial search will be conducted with no restrictions on language, only studies published in English will be included in the QES. The purpose of including non-English publications in the search is to identify the extent of potentially relevant studies that are not in English and whether this may lead to language bias and thus a potential limitation of the QES.

### Study selection

All the citations retrieved from the search will be uploaded to Covidence which is a proprietary software, an open access similar to this software could be used- Rayyan, a web-based software specifically designed to streamline the screening process for reviews. Duplicates will be identified and removed automatically by Covidence. Using AMSTAR 2 guidance stands for "A Measurement Tool to Assess Systematic Reviews" version 2. It's a widely used tool designed to critically evaluate the methodological quality of systematic reviews especially in healthcare ( Shea *et al.,* 2007), two reviewers will initially pilot screen 10% of retrieved records on title and abstract independently to ensure a consistent application of the inclusion criteria. If agreement on include or exclude is more than 80% one reviewer will proceed to further complete the screening by title and abstract. Full-text articles of relevant studies will be independently screened by two reviewers. Any disagreement or conflicts will be resolved through discussion and consensus, involving a third reviewer if necessary. The PRISMA flowchart will be used to report the screening and inclusion process ( Moher *et al.,* 2009).

### Quality appraisal of included studies

The methodological quality of studies included will be appraised by using the Critical Appraisal Skills Programme (CASP) quality assessment tool specifically designed for qualitative research. This assessment tool consists of 10 questions based on three key aspects (i) clarity and validity of research papers (ii) the methodology used, and (iii) the relevance and presentation of the findings. Two reviewers will independently appraise the included studies to assess their overall quality. 

### Data extraction and synthesis

Data will be extracted from the included studies through a purposively designed data extraction form (Appendix 4.) developed for the QES based on the aim of the review. The form will be initially piloted by two reviewers independently on the first three included studies to ensure its suitability; if required amendments will be made to the format and documented in the QES. To ensure the robustness of the process, one reviewer will extract the data, and a second reviewer will independently verify the extracted data. Data extracted will include study aim, study design, source and type of publication (journal paper, unpublished thesis, conference proceeding, abstract, etc.), location and year of study, description of participants, sampling and study settings, method (s) of data collection and analysis, and findings related to the views, experiences and perspectives of fathers on childbirth attendance.

Findings from the included studies will be synthesised using Thomas and Harden’s (2008) three-stage thematic synthesis approach. The extracted ‘findings’ data from included studies will be coded (line by line coding). These codes will then be grouped into descriptive themes, and, from these themes, analytical themes will be developed.

### Assessing the confidence of the QES findings

A systematic assessment of the confidence of the QES findings will be conducted using the Grading of Recommendations Assessment, Development, and Evaluation-Confidence in the Evidence from Reviews of Qualitative research (GRADE-CERQual) framework. The framework focuses primarily on four components: (i) Methodological limitations, (ii) Coherence (iii) Adequacy of data, and (iv) Relevance of the findings ( Booth *et al.*, 2018). To ensure consistency and transparency, a pre-defined set of criteria aligned with the GRADE-CERQual framework will be established prior to commencing the review. Two reviewers will independently evaluate each study and assign confidence ratings. The confidence ratings are categorized into four levels: very low, low, moderate, and high confidence, based on the four primary components. Any discrepancies will be discussed and resolved by consensus.

## Discussion

Undertaking this comprehensive QES will provide insight and in-depth understanding of fathers’ perspectives on childbirth attendance and will inform clinical practice and guidelines for a family-centered approach in maternity care. The findings of the QES will be disseminated through peer-reviewed journals in the field of maternal health, presentations at relevant conference and through posts on social media platforms.

## Data Availability

No data are associated with this article. OSF: Fathers' experiences, views, and perspectives of childbirth attendance: A qualitative evidence synthesis protocol, https://osf.io/9wscm/ This project contains the following underlying data: 1.  Supplementary File 1 - Search Strategy.pdf 2.  Supplementary file 2- PRISMA flow chart.pdf 3.  Supplementary 3- PRESS checklist.pdf 4.  Supplementary 4- Data Extraction form.pdf

## References

[ref-1] Beck CT : The consequences of lack of social support for mothers after childbirth. *Arch Womens Ment Health.* 2009;12(1):39–49.

[ref-2] Booth A Lewin S Glenton C : Applying GRADE-CERQual to qualitative evidence synthesis findings-paper 7: understanding the potential impacts of dissemination bias. *Implement Sci.* 2018;13(Suppl 1): 12. 10.1186/s13012-017-0694-5 29384076 PMC5791043

[ref-3] Chapman L : Searching: expectant father’s experiences during labour and birth. *J Perinat Neonatal Nurs.* 1991;4(4):21–29. 10.1097/00005237-199103000-00006 1993982

[ref-4] Cooke A Smith D Booth A : Beyond PICO: the SPIDER tool for qualitative evidence synthesis. *Qual Health Res.* 2012;22(10):1435–43. 10.1177/1049732312452938 22829486

[ref-5] Davis-Floyd R : Birth as an option: power, participation, and autonomy in prenatal care. Routledge,2009.

[ref-6] Draper J : Whose welfare in the labour room? a discussion of the increasing trend of fathers' birth attendance. *Midwifery.* 1997;13(3):132–8. 10.1016/s0266-6138(97)90003-6 9362853

[ref-7] Grøtela DS Ødegård SH Øien NG : Partner support during childbirth: associations with women's satisfaction with birth and postpartum depression. *Birth.* 2018;45(4):307–316.

[ref-8] Hodnett EL Gates S Hannah M : Continuous support for women during childbirth. *Cochrane Database Syst Rev.* 2014;7(7): CD003766. 10.1002/14651858.CD003766.pub5 23857334

[ref-9] Johnson M Ayers S Lupton M : The effect of Fathers' presence at birth on their emotional well-being and parenting confidence. *Birth.* 2012;39(3):202–210.

[ref-11] Lamb ME Pleck JH Lerner G : The changing nature of fatherhood. *The Future of Children.* 2004;14(1):10–25.

[ref-12] Leap N Murphy S : Fathers and Mothers in the delivery room: changing roles and emotional responses. *Birth.* 1994;21(4):213–220.7857468

[ref-13] Leathers SJ Esterberg KG : On Father's day: a look back at fathers in the birthing room. *BC Med J.* 2004;46(6):322–323. Reference Source

[ref-14] Mathews HJ Macintyre S : International variations in maternity care practices: cultural influences and policy implications. *Women and Birth.* 2018;31(2):142–149.

[ref-24] McNab E Martin CJH Norris G : Factors that influence father’s experiences of childbirth and their implications upon postnatal mental health: a narrative systematic review. *Nurse Educ Pract.* 2022;65: 103460. 10.1016/j.nepr.2022.103460 36244315

[ref-15] Mohammad-Ali MH Mohamad H Othman MS : Cultural and religious factors influencing fathers’ involvement during childbirth: a systematic review. *J Relig Health.* 2022;61(2):723–742.

[ref-16] Moher D Liberati A Tetzlaff J : Preferred reporting Items for systematic reviews and meta-analyses: the PRISMA statement. *Ann Intern Med.* 2009;151(4):264–9, W64. 10.7326/0003-4819-151-4-200908180-00135 19622511

[ref-17] Moore ER Anderson PC : Continuous support for women during childbirth. *N Engl J Med.* 2017;376(7):689–697.

[ref-18] Mprah A Haith-Cooper M Duda-Mikulin E : A systematic review and narrative synthesis of fathers’ (including migrant fathers’) experiences of pregnancy and childbirth. *BMC Pregnancy Childbirth.* 2023;23(1): 238. 10.1186/s12884-023-05568-8 37041486 PMC10088224

[ref-19] Schmied V Wegerer A Abels S : Partner support during childbirth and postpartum depression: a systematic review and meta-analysis. *Arch Womens Ment Health.* 2018;21(2):131–143.

[ref-20] Shea BJ Grimshaw JM Wells GA : Development of AMSTAR: a measurement tool to assess the methodological quality of systematic reviews. *BMC Med Res Methodol.* 2007;7: 10. 10.1186/1471-2288-7-10 17302989 PMC1810543

[ref-21] Thomas J Harden A : Methods for the thematic synthesis of qualitative research in systematic reviews. *BMC Med Res Methodol.* 2008;8: 45. 10.1186/1471-2288-8-45 18616818 PMC2478656

[ref-22] World Health Organization [WHO]: Appropriate technology for birth. *Lancet.* 1985;24(8452):436–437. 2863457

[ref-23] WHO: WHO recommendations on health promotion interventions for maternal and newborn health. Geneva, World Health Organization,2015. Reference Source 26180864

