# Supplementary material for: Fathers' experiences, views and perspectives of childbirth attendance: A qualitative evidence synthesis protocol
Source: HRB Open Res. 2024 Jun 6;7:34. [Version 1] doi: 10.12688/hrbopenres.13879.1 (PMC11318514; doi:10.12688/hrbopenres.13879.1)
Supplement: Peer review history

# Reviewer report: Reviewer response for version 1

**DOI:** 10.21956/hrbopenres.15216.r40585

**Published:** 2024-08-07

**Authors:** Rakime Elmir

**Affiliations:** 1 School of Nursing and Midwifery, University of Western Sydney, Sydney, Australia

Thank you for the opportunity to review this protocol titled 'Fathers experiences, views and perspectives of childbirth attendance: A qualitative evidence synthesis protocol. 

 The abstract provides an overview of the protocol and justifies the needs for the study.  A comprehensive introduction presenting the historically nature of men's presence at birth is presented. A gap in the literature is clearly articulate. 

 I would suggest rewording qualitative evidence synthesis to just qualitative synthesis.

 The inclusion and exclusion criteria is appropriate, however a limit to the date of published papers that will be reviewed needs to be placed and justified to assist in containing the search

Is the study design appropriate for the research question?

Yes

Is the rationale for, and objectives of, the study clearly described?

Yes

Are sufficient details of the methods provided to allow replication by others?

Yes

Are the datasets clearly presented in a useable and accessible format?

Yes

Reviewer Expertise:

Women's health, midwifery

I confirm that I have read this submission and believe that I have an appropriate level of expertise to confirm that it is of an acceptable scientific standard.

# Reviewer report: Reviewer response for version 1

**DOI:** 10.21956/hrbopenres.15216.r40882

**Published:** 2024-07-13

**Authors:** Maria Noonan

**Affiliations:** 1 Department of Nursing and Midwifery, University of Limerick, Limerick, County Limerick, Ireland

Thank you for asking me to review this comprehensive, clearly rationalised and well-written QES protocol: *Fathers' experiences, views and perspectives of childbirth attendance: A qualitative evidence synthesis protocol*. I look forward to reading the final article.

 The methods for searching, study selection, quality appraisal, synthesis and assessment of confidence in review findings are well described, enable replication and align with published guidance on conduct of QES.

 I suggest the following minor changes:

 Abstract: Include 'grey literature sources' to be searched.

 Search strategy: Consider translating and including non-English publications identified in the search strategy.

 Study selection: Suggest consistency across the protocols (Prospero and HRB Open Research) and consider two reviewers independently undertake title and abstract screening.

 Quality appraisal of included studies: Suggest authors specify how disagreements between reviewers will be resolved in relation to the quality appraisal of included studies.

 Data extraction and synthesis: Please indicate whether findings from the included studies will be extracted from the findings/results section of the included studies or from both the findings/results section and discussion section.

 Consider including an author reflexivity statement in the protocol.

 Consider completing the most recent version of the Preferred Reporting Items for Systematic Reviews and Meta-analyses (PRISMA) flow diagram (Page et al., 2021).

Is the study design appropriate for the research question?

Yes

Is the rationale for, and objectives of, the study clearly described?

Yes

Are sufficient details of the methods provided to allow replication by others?

Yes

Are the datasets clearly presented in a useable and accessible format?

Yes

Reviewer Expertise:

I have experience of conducting and publishing primary qualitative research studies and QES.

I confirm that I have read this submission and believe that I have an appropriate level of expertise to confirm that it is of an acceptable scientific standard.

# Reviewer report: Reviewer response for version 1

**DOI:** 10.21956/hrbopenres.15216.r40880

**Published:** 2024-07-04

**Authors:** Christine Margaret Furber

**Affiliations:** 1 University of Salford, Salford, England, UK

The protocol is comprehensive. It is clear and easy to follow. 

 I noted that you had inclusion after 18years of age and wondered if you had thought of younger fathers? 

 I think you need to add observation to the design methods.  

 The measures in place for rigour in the assessments. 

 Supplementary materials are also suitable.

Is the study design appropriate for the research question?

Yes

Is the rationale for, and objectives of, the study clearly described?

Yes

Are sufficient details of the methods provided to allow replication by others?

Partly

Are the datasets clearly presented in a useable and accessible format?

Yes

Reviewer Expertise:

qualitative methods

I confirm that I have read this submission and believe that I have an appropriate level of expertise to confirm that it is of an acceptable scientific standard.
